# Psychoeducation versus psychoeducation integrated with yoga for family caregivers of people with Alzheimer's disease: a randomized clinical trial

**DOI:** 10.1007/s10433-023-00792-9

**Published:** 2023-11-25

**Authors:** Edivaldo Lima de Araujo, Marcos Rojo Rodrigues, Elisa Harumi Kozasa, Shirley Silva Lacerda

**Affiliations:** 1 Faculdade Israelita de Ciências da Saúde Albert Einstein, Hospital Israelita Albert Einstein, São Paulo, Brazil; 2 Instituto de Ensino e Pesquisa em Yoga, São Paulo, Brazil; 3 Hospital Israelita Albert Einstein, São Paulo, Brazil

**Keywords:** Alzheimer’s disease, Burden, Family caregivers, Psychoeducation, Quality of life, Yoga

## Abstract

We evaluate and compare the effectiveness of two psychoeducation programs on the burden and quality of life of family caregivers of people with Alzheimer's disease: one of psychoeducation alone and the other integrated with yoga. Forty-nine participants were randomly allocated to two groups, 25 to the psychoeducation integrated with yoga group (G1) and 24 to the psychoeducation group (G2). The programs took place online through Google Meet and consisted of eight weekly meetings, lasting 30 min for psychoeducation and 30 min for yoga. Participants were evaluated by the Burden Interview Scale (BI-Zarit), Quality of Life Scale in Alzheimer's Disease-Caregiver Version (CQOL-AD), Depression, Anxiety and Stress Scale (DASS-21), Mindfulness and Awareness Scale (MAAS) and a satisfaction survey. Both groups showed reduced overload according to the BI-Zarit scale (*F*_(1)_ = 4.435, *p* = 0.041, *η*^2^_*p*_ = 0.086) and improvement in the domains of physical health (*F*_(1)_ = 4.881, *p* = 0.032, *η*^2^_*p*_ = 0.094), memory (*F*_(1)_ = 4.192, *p* = 0.046, *η*^2^_*p*_ = 0.082) and money (*F*_(1)_ = 4.862, *p* = 0.032, *η*^2^_*p*_ = 0.094) in the CQOL-AD. We detected a significantly higher improvement of G1 in memory (*F*_(1)_ = 4.192, *p* = 0.046 *η*^2^_*p*_ = 0.082), money (*F*_(1)_ = 7.147, *p* = 0.010, *η*^2^_*p*_ = 0.132) and friends (*F*_(1)_ = 4.828, *p* = 0.033, *η*^2^_*p*_ = 0.093). The G1 when compared to the G2 did not demonstrate significant effects of the BI-Zarit, in the total scores CQOL-AD, MAAS and DASS-21. The study showed that both psychoeducation alone and integrated with yoga reduced the burden of family caregivers of people with Alzheimer's disease, and that the integration of online yoga practice with psychoeducation potentiated the improvement only in some aspects of the quality of life and subjective perception of burden reduction.

*Clinical trial registration* https://ensaiosclinicos.gov.br/, identifier RBR-794593r, retrospectively registered.

**Supplementary Information:**

The online version contains supplementary material available at 10.1007/s10433-023-00792-9.

## Introduction

Population aging is a worldwide phenomenon and, associated with this process, an increase in chronic degenerative diseases such as dementia is expected (ALZINT 2020). It is currently estimated that there are 55 million people living with dementia in the world. The projection for 2050 is 139 million (ALZINT 2020), a number that takes epidemic proportions with great socioeconomic impact, from both the individual and community point of view.

Alzheimer's disease (AD) dementia is the most prevalent in the world, ranging from 53 to 75% of cases (Ayodele et al. 2021). It is progressive and, so far, irreversible. With the evolution of dementia, in addition to the decline in cognitive functions there is a progressive impairment of the physical skills necessary for the maintenance of activities of daily living, initially for the most complex, and in the more advanced stages, basic activities such as eating and bathing. Inevitably, neuropsychiatric symptoms of dementia (NSD) such as depression, anxiety, delusions, hallucinations, agitation, aggression, insomnia, wandering and apathy will begin to manifest as dementia progresses. The more frequent and intense the NSD, the greater the caregiver burden and the chances of early institutionalization of elderly people with dementia (Bessey and Walaszek 2019; Chiu et al. 2013).

In this context, more and more family members become caregivers and are faced with a growing demand for care, because as the disease progresses, the patient's dependence increases and gradually requires more time dedicated to care. Therefore, many family members are forced to reshape their own lives to accommodate the patient's current state and his/her future to the detriment of their own life projects (ALZINT 2020; Ayodele et al. 2021).

The experience of caring for dependent elderly people is pointed out by family caregivers as a task that causes stress and exhaustion (Diniz et al. 2018; Win et al. 2017). It is estimated that between 60 and 70% of family caregivers of patients with dementia have medical and/or psychiatric problems, including anxiety, depression, sleep disorders, abusive use of alcohol and psychotropic medications, as well as increased rates of morbidity and mortality (Pessotti et al. 2018; Liu et al 2017).

The burden on family caregivers can increase due to the lack of information about the disease that meets their own needs and those of the person with dementia. Therefore, psychoeducation has been presented as a strategy to help them, since understanding what the disease is, its evolution, how to deal with the functional decline and NSD, how to understand the sick family member and their own feelings can facilitate their daily routine. In a literature review on interventions for those caregivers, Cheng et al. (2019) found that psychoeducation and multi-component programs based on psychotherapeutic principles have been widely used to support them. More recently, Cravello et al. (2021) demonstrated that a course to support caregivers of people with dementia and cognitive decline reduced the burden of care, even in an atypical and negative period such as the lockdown in the COVID-19 pandemic.

The literature shows growing evidence supporting the role of yoga and meditation as non-pharmacological strategies, in addition to knowledge of the disease, for improving caregivers' mood and sleep disorders (Danucalov et al. 2017). Currie et al. (2022), who evaluated the effect of an intervention based on the Yoga of Immortals app on the mental health of healthcare professionals, found reduced stress, anxiety and depression and improved sleep quality. In a controlled pilot study that used an integrated yoga intervention for professional caregivers of people with Alzheimer's disease, Chhugani et al. (2018) found similar results. Parkman and Olausson (2023) carried out an integrative review on the effectiveness of yoga for caregivers of people with dementia, demonstrating that yoga can be useful in reducing stress, depression and anxiety and can also improve quality of life, self-compassion, mindfulness, sleep quality and diastolic blood pressure of caregivers.

Studies that associate psychoeducation and yoga are scarce in the literature. Donnelly et al. (2020), in a qualitative study of LoveYourBrain Yoga, evaluated an intervention based on breathing exercises, yoga, meditation and psychoeducation and found that people with brain injury had better community integration and benefits in physical, psychological and social health.

Considering that interventions based on psychoeducation and yoga can reduce the burden and improve the quality of life of family caregivers, the objective of this study was to evaluate the effectiveness of a psychoeducation program, and another of psychoeducation integrated with yoga, on the burden and quality of life of family caregivers of people with AD. We assume that after carrying out the programs, these individuals can better deal with the disease and symptoms of their family member, have their burden reduced and, consequently, be able to improve their quality of life.

## Methods

We carried out a randomized clinical trial between the first and second semesters of 2022 comparing two groups of family caregivers of people diagnosed with Alzheimer's disease. Participants lived in the State of Minas Gerais, Brazil.

### Participants

The sample comprised 197 family members of people with AD, assisted in a public health care program for the elderly and private medical offices, who were recruited by telephone and social media, 58 of whom were eligible for the study. The inclusion criteria were being a family caregiver responsible for the direct care of the demented elderly; not receiving payment for the care; having dedicated themselves to the care at least eight hours a day for at least three months and having access to the Internet. Exclusion criteria were caregivers under 18 years of age; those unable to understand and/or answer the scales; participants with severe or limiting chronic diseases; those who were not available to participate in meetings; yoga practitioners and experienced meditators; those who already had a family member participating in the study; and those who cared for elderly who met Clinical Dementia Rating (CDR) 0 and 0.5 (no dementia and questionable dementia).

**Fig. 1:**
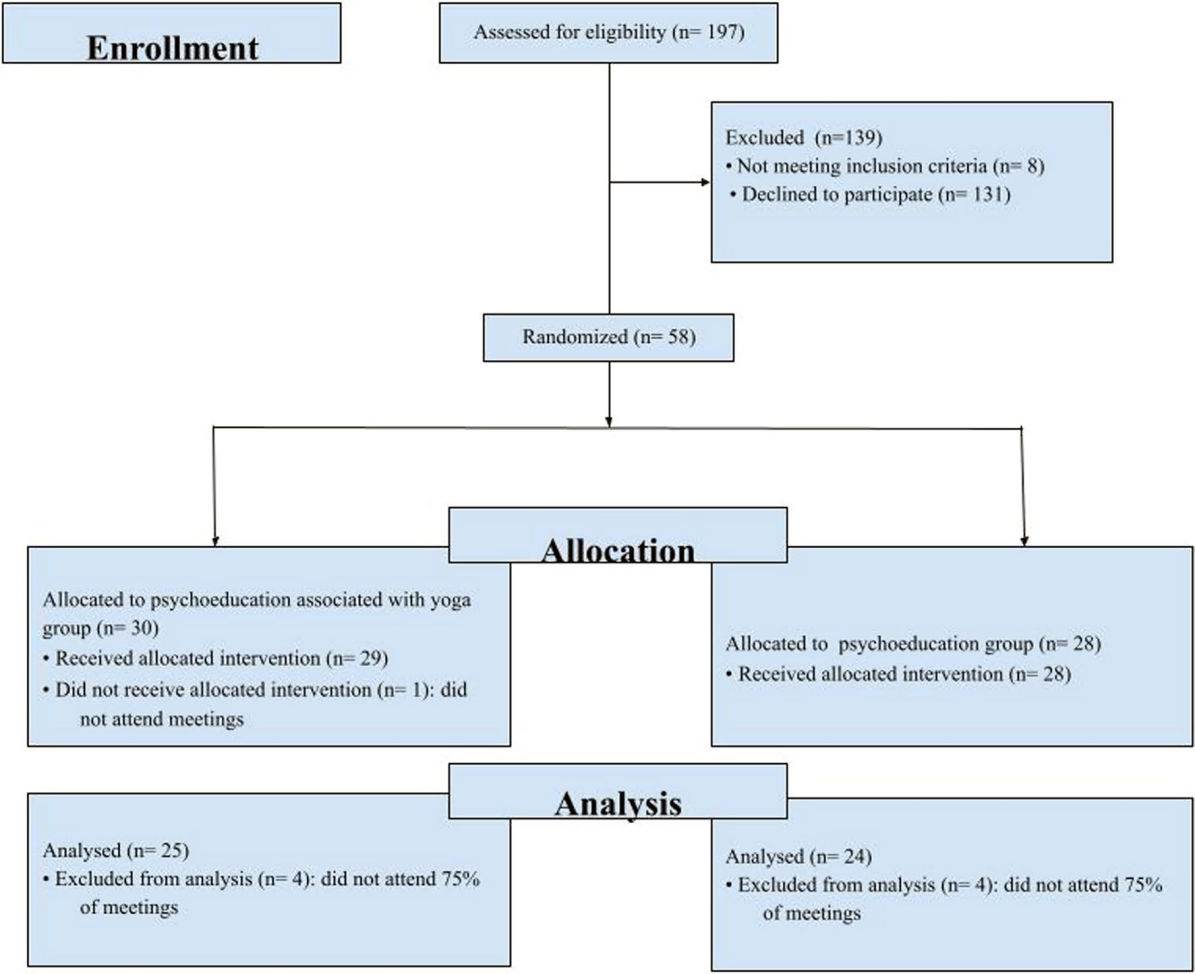
Participant allocation flow-chart

Participants were randomly assigned to two groups, using a randomization list generated on the randomizer.org: psychoeducation group integrated with yoga (G1) and psychoeducation group (G2). Eight participants, four in each group, were excluded because they had not participated in at least 75% of the meetings, and one participant did not attend any of the meetings. The final sample consisted of 49 participants, 25 from G1 (mean age = 54.96 ± 9.36) and 24 from the G2 (mean age = 53.54 ± 8.48). The two groups did not differ significantly from each other regarding age; years of schooling; marital status; relationship; hours dedicated to care; family income; and severity of dementia (*p* > 0.05) (Fig. 1).

## Instruments

### Sociodemographic form

The sociodemographic form included questions about age; sex; marital status; degree of kinship with the demented person; number of hours dedicated to care; whether they were the primary caregiver; schooling; and family income.

## Clinical-epidemiological form

The clinical-epidemiological form included questions about health conditions; use of medication; alcohol use; tobacco use; physical activity; and leisure activities.

### Burden Interview Scale (BI-Zarit)

It assesses caregiver burden. With an Alpha Cronbach coefficient of 0.87, the Brazilian version contains 22 items related to health; social and personal life; financial situation; well-being; and personal relationships. Each question has four response options with scores ranging from 0 (never) to 4 (always), and the higher the score, the greater the overload (Scazufca 2002).

### Alzheimer's Disease Quality of Life Scale, caregiver version (CQOL-AD)

The Brazilian version consists of 13 items that can score four points each, ranging from poor (1 point) to excellent (4 points). The higher the total score, the better the quality of life. It addresses the domains health, mood, living situation, memory, family, marriage, friends, self, ability to do chores, ability to do things for fun, money and life as a whole. We asked the participants to evaluate how important each of those domains is for their quality of life and then how they see their present situation regarding each domain. The final score is the addition of points of all the items of their present situation. The Alpha Cronbach coefficient of this version is 0.86 (Novelli 2006).

### Depression, Anxiety and Stress Scale (DASS-21)

It consists of 21 Likert-type questions with four response options (0–3). Each subscale assesses the depression, anxiety and stress domains. The total for each domain ranges from 0 to 21, and the final score is multiplied by 2. The final score of each subscale suggests classification as normal, mild, moderate, severe or extremely severe. Items 3, 5, 10, 13, 16, 17 and 21 correspond to the depression domain and scores 0–9 suggest absence (normal) of depression, 10–13 mild depression, 14–20 moderate depression, 21–27 severe depression and above 28 extremely severe depression. Anxiety corresponds to items 2, 4, 7, 9, 15, 19 and 20, and scores 0–7 suggest absence of anxiety (normal), 8 and 9 mild anxiety, 10–14 moderate anxiety, 15–19 severe anxiety and above 20 extremely severe anxiety. Finally, stress corresponds to items 1, 6, 8, 11, 12, 14 and 18 and scores from 0 to 14 suggest normal result (absence of stress), from 15 to 18 mild stress, 19–25 moderate, 26–33 severe and above 34 extremely severe stress. The Brazilian version has an Alpha Cronbach coefficient of 0.92 for depression, 0.86 for anxiety and 0.90 for stress (Vignola and Tucci 2014).

### Mindfulness and Awareness Scale (MAAS)

It assesses self-reported attention focused on present-moment awareness. With an Alpha Cronbach coefficient of 0.83, the Brazilian version consists of 15 items on a six-point Likert scale, ranging from 1 (almost always) to 6 (seldom). The minimum score is 15 points, and the maximum is 90, indicating the minimum and maximum level of mindfulness and awareness, respectively (Barros et al. 2015).

### Clinical Dementia Rating Scale (CDR)

It allows evaluating the staging of dementia based on global cognitive and functional assessment. Subjects are classified into the following categories: 0 = no dementia, 0.5 = questionable dementia, 1 = mild dementia, 2 = moderate dementia, and 3 = advanced dementia. The Brazilian version showed strong agreement between clinical diagnosis and dementia (*k* = 0.93). Regarding the criteria, it presented 91.2% sensitivity, 100% specificity, positive predictive value of 100%, negative predictive value of 97.6% and accuracy of 98.1% (Montaño and Ramos 2005).

### Satisfaction survey

It evaluated the subjective perception of satisfaction with the program, whether the participant was able to better deal with the disease and symptoms of the demented family member, whether there was a reduction in their burden, and whether there was an improvement in their quality of life. Four 4-point Likert-type questions were asked, ranging from 1 (minimum) to 4 (maximum). The higher the score, the greater the perception of improvement.

All assessments at T0 and T1 were carried out in person by the researcher. There was no blinding of the researchers.

### Interventions

The psychoeducation program was carried out in eight 30-min virtual weekly meetings through Google Meet. For G1 we applied the same psychoeducation program and 30 min of yoga. Two WhatsApp groups managed by the researcher were created, aiming to encourage participation in the meetings. In addition, we sent videos to G1 encouraging the practice of yoga at least twice a week and a calendar for them to register the days they practiced.

Our psychoeducation program provided information about the disease, non-pharmacological strategies to deal with NSD, guidelines on how to recognize the signs of overload, as well as strategies to reduce this overload and improve quality of life.

The yoga exercises chosen for this work originated from the system known as *Hatha Yoga* and among the techniques known in this system we highlighted the *Asanas* for our work.

The postures chosen were adapted to the specific conditions of an audience with little affinity for yoga and, in many cases, for physical activity in general. Although we had to adjust the exercises, we did not compromise the principles that guide a traditional yoga practice. This was done to facilitate adherence and involvement of practitioners.

The classes were conducted as follows:

- Classes 1 and 2: *Asanas—*20 min of exercises done in a sitting position (on chairs); relaxation—5 min (on chairs); and meditation—5 min (on chairs).
- Classes 3, 4 and 5: *Asanas—*20 min divided into exercises in standing and sitting positions (standing and on chairs); relaxation—5 min (on chairs); and meditation—5 min (on chairs).
- Classes 6, 7 and 8: *Asanas—*20 min (lying down); relaxation—10 min (lying down).

The yoga program classes are detailed in Supplementary Information.

In addition to the synchronous online classes, we recorded videos that were sent to the participants and encouraged repetition of the practice during the week.

**Fig. 2:**
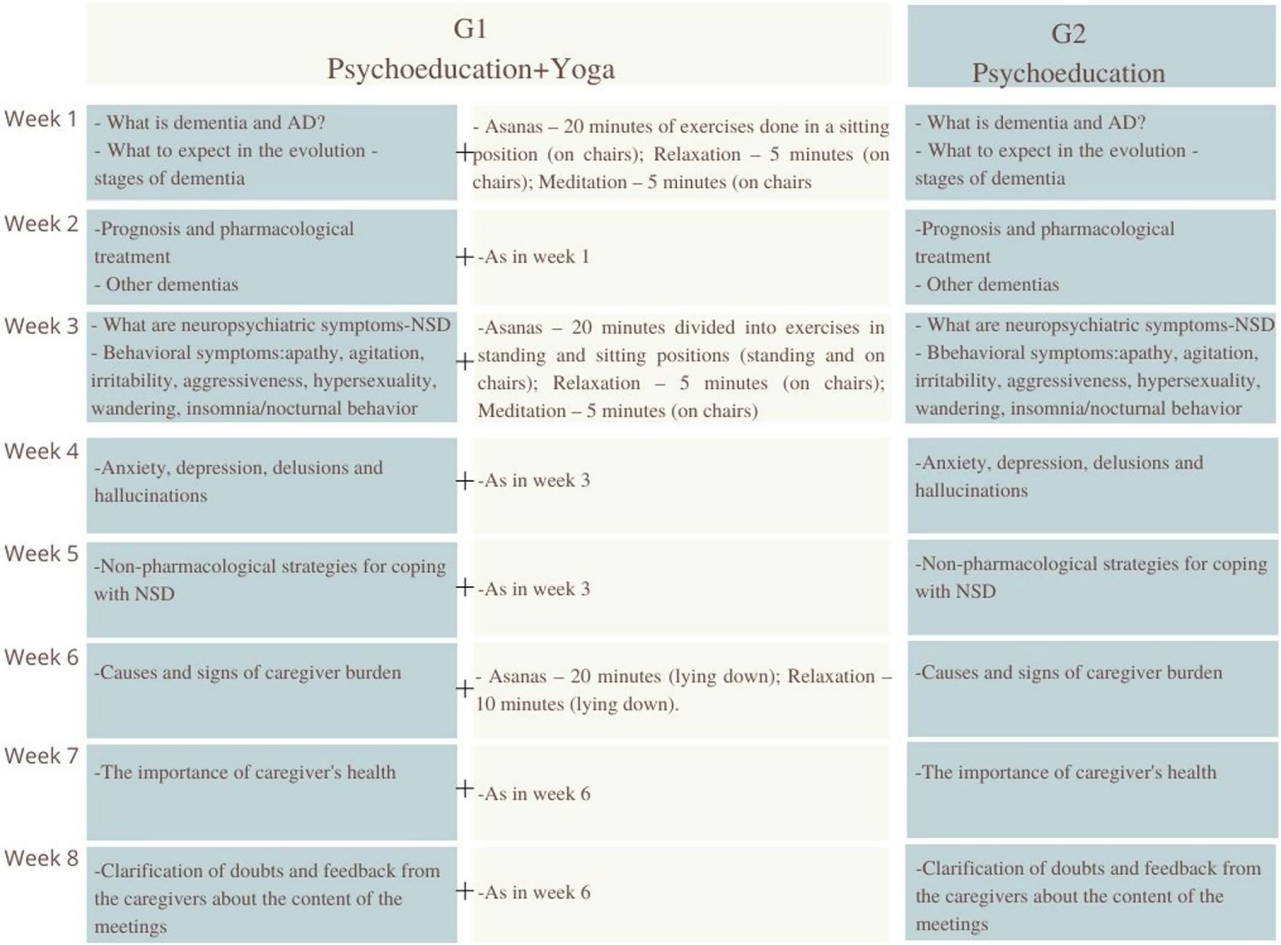
Interventions design

The intervention design is shown in Fig. 2.

### Statistical analysis

Group differences were evaluated by the chi-squared and Student's t tests, and the generalized linear mixed model (GLMM) was used to compare scores between and within groups at baseline (T0) and 8 weeks after the intervention (T1)(Cohen 1973). All analyses were performed using the JASP TEAM program (2022, version 0.16.4).

## Results

All participants were female, most of them being daughters, 80% in G1 and 75% in G2. In G1, the mean age was 54.96 (± 9.36) years and in G2, 53.54 (± 8.48) years. The two groups did not differ significantly in terms of age; years of schooling; marital status, degree of kinship with the demented person; hours devoted to care; family income; and severity of CDR dementia (*p* > 0.05). Detailed sociodemographic characteristics are presented in Table 1.

**Table 1:** Demographics variables

	G1 (*n* = 25)		G2 (*n* = 24)		*p* value^a^
	Mean	sd	Mean	sd	
Age (years)	54.96	9.36	53.54	8.48	0.587
CDR	2.24	0.72	2.08	0.58	0.409

	*n*	%	*n*	%	*p* value^b^
Years of schooling					
Less than 8 years	4	16.00	7	29.17	0.359
Between 8 and 12 years	8	32.00	9	37.50	
More than 12 years	13	52.00	8	33.33	
Marital status					
Single	1	4.00	2	8.33	0.874
Married	18	72.00	15	62.50	
Widow	2	8.00	2	8.33	
Divorced	4	16.00	5	20.83	
Relationship					
Daughter	20	80.00	18	75.00	0.405
Wife	3	12.00	3	12.50	
Friend	1	4.00	0	0	
Niece	0	0	1	4.17	
Daughter in law	1	40.00	0	0	
Sister	0	0	2	8.33	
Hours dedicated to care					
8 h	8	32.00	4	16.67	0.278
8–12 h	3	12.00	5	20.83	
12–16 h	0	0	2	8.33	
16–24 h	14	56.00	13	54.17	
Family income (minimum wage)					
Less than 2	5	20.00	10	41.67	0.378
2–4	11	44.00	6	25.00	
4–10	5	20.00	6	25.00	
10–20	3	12.00	1	4.17	
More than 20	1	4.00	1	4.17	

^a^Student *t* test; ^b^Chi-squared test

At T1, both groups showed reduced overload according to the BI-Zarit scale (*F*_(1)_ = 4.435, *p* = 0.041, *η*^2^_*p*_ = 0.086). Additionally, there was no difference between them (*F*_(1)_ = 0.316, *p* = 0.577, *η*^2^_*p*_ = 0.007). In the CQOL-AD scale, there were no differences in the total scores; however, improvement was detected in the domains of physical health (*F*_(1)_ = 4.881, *p* = 0.032, *η*^2^_*p*_ = 0.094), memory (*F*_(1)_ = 4.192, *p* = 0.046, *η*^2^_*p*_ = 0.082) and money (*F*_(1)_ = 4.862, *p* = 0.032, *η*^2^_*p*_ = 0.094) in both groups. Comparing the groups, we detected a significantly higher improvement of G1 in memory (*F*_(1)_ = 4.192, *p* = 0.046 *η*^2^_*p*_ = 0.082) and money (*F*_(1)_ = 7.147, *p* = 0.010, *η*^2^_*p*_ = 0.132). In the friends domain, there was a difference between the groups (*F*_(1)_ = 4.828, *p* = 0.033, *η*^2^_*p*_ = 0.093), but the statistical difference must be related to the low valuation of G2 at T1. The G1 when compared to the G2 did not demonstrate significant effects on most of the primary outcomes, including BI-Zarit, MAAS and DASS-21 (Table 2).

**Table 2:** Comparisons between G1 and G2 for assessment of burden, attention, depression, anxiety, stress and quality of life in T0 (baseline) and after 8 weeks (T1)

	G1 (*n* = 25)		G2 (*n* = 24)		Time effect^a^ *p* (*η*^2^_*p*_)	Group effect^a^ *p* (*η*^2^_*p*_)	Time*group effect^a^ *p* (*η*^2^_*p*_)
	T0	T1	T0	T1			
	Mean (sd)	Mean (sd)	Mean (sd)	Mean (sd)			
BI-ZARIT	39.92 (13.08)	35.96 (14.42)	44.38 (18.91)	42.08 (15.40)	0.041* (0.09)	0.213 (0.03)	0.577 (0.01)
MAAS	58.36 (14.41)	59.88 (13.13)	56.04 (13.65)	57.17 (15.24)	0.470 (0.01)	0.489 (0.01)	0.914 (0.01)
DASS 21							
Depression	12.56 (9.97)	11.04 (9.73)	12.75 (9.97)	14.08 (11.55)	0.170 (0.04)	0.264 (0.03)	0.949 (0.01)
Anxiety	10.96 (8.36)	10.96 (10.36)	11.92 (10.27)	11.83 (11.57)	0.964 (0.01)	0.742 (0.01)	0.964 (0.01)
Stress	17.52 (9.13)	16.64 (8.06)	18.00 (12.17)	19.50 (11.53)	0.805 (0.01)	0.535 (0.01)	0.345 (0.02)
CQOL-AD							
Physical health	2.44 (9.96)	2.60 (0.76)	2.21 (0.72)	2.54 (0.66)	0.032* (0.09)	0.461 (0.01)	0.441 (0.01)
Energy	2.36 (0.70)	2.60 (0.82)	2.29 (0.81)	2.42 (0.65)	0.100 (0.06)	0.497 (0.01)	0.599 (0.01)
Mood	2.52 (0.71)	2.44 (0.77)	2.50 (0.88)	2.75 (0.79)	0.370 (0.02)	0.484 (0.01)	0.086 (0.06)
Living situation	3.32 (0.56)	3.36 (0.64)	3.17 (0.76)	3.08 (0.580	0.842 (0.01)	0.151 (0.04)	0.570 (0.01)
Memory	2.76 (0.66)	2.76 (0.58)	2.21 (0.78)	2.65 (0.58)	0.046* (0.08)	0.035* (0.09)	0.046* (0.08)
Family	3.32 (0.63)	3.20 (0.76)	2.96 (0.81)	3.04 (0.81)	0.847 (0.01)	0.186 (0.04)	0.287 (0.02)
Marriage	3.05 (0.76)	3.20 (0.77)	2.64 (1.08)	2.87 (1.03)	0.110 (0.08)	0.207 (0.05)	0.773 (0.01)
Friends	2.72 (0.84)	2.88 (0.67)	2.92 (0.65)	2.63 (0.71)	0.525 (0.01)	0.871 (0.01)	0.033* (0.09)
Self as a whole	2.56 (0.71)	2.72 (0.78)	2.54 (0.83)	2.54 (0.78)	0.387 (0.02)	0.607 (0.01)	0.387 (0.02)
Ability to do chores	2.88 (0.78)	2.72 (0.79)	2.67 (0.76)	2.79 (0.83)	0.897 (0.01)	0.698 (0.01)	0.297 (0.02)
Ability to do things for fun	2.52 (0.92)	2.32 (0.90)	2.04 (0.86)	2.12 (0.90)	0.688 (0.01)	0.118 (0.05)	0.331 (0.02)
Money	2.72 (0.61)	2.68 (0.69)	2.15 (0.45)	2.54 (0.59)	0.032* (0.09)	0.016* (0.12)	0.010* (0.13)
Life as a whole	2.84 (0.62)	2.88 (0.60)	2.71 (0.81)	2.75 (0.53)	0.691 (0.01)	0.402 (0.02)	0.994 (0.01)
Total score	35.40 (5.52)	35.92 (5.88)	32.04 (5.54)	33.92 (5.83)	0.053 (0.08)	0.082 (0.06)	0.267 (0.03)

^a^GLMM, *p < 0.05, Effect size η^2^_p_ (Less than 0.01 indicates a small effect size, 0.06 indicates a medium effect size and greater than 0.14 indicates a large effect size) (Cohen 1973)

In the evaluation of the subjective perception of satisfaction with the programs, most participants were satisfied or very satisfied, G1 = 100% (*n* = 25), G2 = 95.83% (*n* = 23). As for specific aspects, they had the perception of being able to deal better or much better with the disease and the symptoms of the family member G1 = 82.6% (*n* = 20); G2 = 75% (*n* = 18); felt their burden was reduced or greatly reduced G1 = 82.6% (*n* = 20), G2 = 50% (*n* = 11); and observed an improvement or a lot of improvement in their quality of life G1 = 91.3% (*n* = 22), G2 = 66.7% (*n* = 15). In their perception of burden reduction, there was a significant difference between the groups, which was stronger in the G1 (*χ*^2^_(3)_ = 9.074, *p* = 0.028) (Table 3).

**Table 3:** Satisfaction research

	G1 (*n* = 25)		G2 (*n* = 24)		*p* value^a^
	*n*	%	*n*	%	
How satisfied are you with the program?					
Dissatisfied	0	0	1	4.17	0.246
Satisfied	4	16.00	1	4.17	
Very satisfied	21	84.00	22	91.67	
Are you better able to cope with the disease?					
Can handle a little better	5	20.00	6	25.00	0.138
Can handle	13	52.00	6	25.00	
Can handle very well	7	28.00	12	50.00	
Has your burden been reduced?					
No reduction	1	4.00	1	4.17	0.028*
A little reduction	4	16.00	12	50.00	
There was a reduction	15	60.00	5	20.83	
A lot of reduction	5	20.00	6	25.00	
Has your quality of life improved?					
A little improvement	3	12.00	9	37.50	0.085
There was improvement	16	64.00	9	37.50	
A lot of improvement	6	24.00	6	25.00	

*p < 0.05; ^a^Chi-square test

## Discussion

This is a pioneer study in evaluating and comparing the effectiveness of an isolated psychoeducation program and another one, integrated with yoga, on the burden and quality of life of family caregivers of people with Alzheimer's disease.

Interest in those issues has been growing due to the aging of the global population and, as a result, the increase in the global burden of the disease. 

All participants in this study were female, which coincides with current literature (Alzheimer’s Association 2022; Kim et al. 2021). However, this fact may have been generated by selection bias with implications for the results.

As in previous studies (Alzheimer’s Association 2022; Grabher 2018; Kim et al. 2021), we observed that most caregivers were married, daughters and primary caregivers. It is assumed that they are people with multiple responsibilities such as taking care of the house, children, and the marital relationship. When the function of caring for a dependent family member is added, their physical, mental and financial burden can increase.

The act of caring for people with dementia such as AD is challenging, due to the series of symptoms that may arise over the course of the disease. In addition to cognitive decline and physical dependence, NSD can contribute to caregivers' physical and mental exhaustion, making them more susceptible to illness (Liu et al. 2017; Grabher 2018; Kim et al. 2021). We did not find differences in the overload of caregivers over the different stages of the patient’s disease, which contrasts with reports in the literature (Koca et al. 2017; Ohno et al. 2021). This result might be explained by the homogeneity of our sample, since most of the elderly fit in CDR = 2 (moderate dementia) and only seven in CDR = 1 (mild dementia).

We found several studies showing that psychoeducation, based on educational approaches to the disease and the development of skills to carry out the exhausting task of caring for those patients, has great potential in reducing caregiver burden (Chiu et al. 2013; Cravello et al. 2021; Cheng et al. 2020; Carnahan et al. 2020; Soong et al. 2020). Our results confirm this potential, as both groups had access to psychoeducation and achieved a significant reduction in the burden. Tawfik et al. (2021), like us, used the BI-Zarit scale to assess caregiver burden in a randomized clinical trial and demonstrated that an 8-session psychoeducational program was effective in reducing caregiver burden caregiver.

In our psychoeducation program, we presented classes on what Alzheimer's disease is, its evolution, treatment and prognosis. Moreover, we described the main behavioral and psychological symptoms of dementias, as evidence indicates that NSD are major causes of caregiver burden (Kim et al. 2021; Chen et al. 2017). However, our main objective was to show caregivers how to recognize the signs of overload and show them non-pharmacological strategies to deal with the NSD, reduce the overload and improve their quality of life.

Evidence demonstrates the effectiveness strategies other than psychoeducation to support caregivers, including yoga. Studies have shown that such intervention can reduce symptoms of depression, anxiety and stress and improve attention and sleep quality (Danucalov et al. 2017; Currie et al.^2022^; Verma et al. 2021). Chhugani et al. (2018) conducted a controlled pilot study to evaluate the effects of yoga on anxiety, depression, stress, and sleep quality of professional caregivers of people with Alzheimer's disease and found that an integrated yoga intervention improved anxiety, depression, stress and sleep quality among caregivers. Consequently, it can facilitate the management of patients’ dementia symptoms, reducing the burden and improving the quality of life of caregivers (Park et al. 2020). In this study, online yoga practice did not yield these same positive results in depression, anxiety, stress and mindfulness. This fact may have happened because it is a population with little affinity for physical activity. Moreover, some participants are not familiar with the online technology, which may have made it difficult for them to understand and carry out the practices properly. In addition, the time and number of weekly classes may have been insufficient to produce the expected effect.

In our quality-of-life assessment, we used the CQOL-AD scale, which addresses several domains by asking the participants how they see each domain in their current situation. The answers range from poor (1) to excellent (4), and the higher the score, the better the quality of life. We did not detect improvement in the global assessment of quality of life, but in the evaluation by domains there was significant improvement in physical health, memory and money in both groups. In the group that practiced yoga, there was higher improvement in the memory and money domains, in addition to the *friends* domain. This difference may have happened since mind–body approaches, such as yoga, aim to improve the level of attention and the appreciation of the present moment, of people and of oneself, as opposed to external and material stressors.

To assess the subjective perception of burden reduction in our satisfaction survey, we used the question: “After the program, do you believe that your burden has been reduced?” There were four response options, ranging from “There was no reduction in burden” to “It greatly reduced burden” and the higher the score, the greater the reduction. We noticed a more significant reduction in the group that, in addition to psychoeducation, had online yoga classes, even though this result was not confirmed by the BI-Zarit scale. This scale also includes the objective assessment of the burden on the caregiver’s personal life resulting from providing care, and the implications for the relationship between the elderly and the caregiver. The scale has 22 questions, the last question being about how much he/she feels overwhelmed. Responses range from never (0) to always (4) and the higher the score, the more severe the overload. The difference in the result between the subjective satisfaction survey and the BI-Zarit scale may have occurred since the practice of yoga provides physical and mental well-being and, consequently, a feeling of relief from the burden of care. The BI-Zarit scale, on the other hand, may have yielded higher scores (greater burden) because it also assesses aspects of objective burden, such as the financial situation, for example.

With the social isolation recently imposed by the COVID-19 pandemic, online support alternatives were implemented to assist the caregiver (Romero-Mas et al. 2021; Leng et al. 2020; Ferretti et al. 2021). A pilot study carried out in São Paulo, Brazil, showed that it is possible to obtain positive results with distance education actions in the management of dementia (Ferretti et al. 2021). In the same pandemic period, an English randomized controlled trial found out that an online yoga program reduced stress and improved well-being for people working from home (Wadhen and Cartwright 2021). We believe that this modality favored the participation of volunteers in our study, as most of whom were daughters and primary caregivers who would probably have difficulty attending face-to-face meetings.

The possible limitations of this study were the online teaching modality of yoga, as it restricts the observation and correction of students' postures; it was not possible to control some variables such as monitor size, sound effect and internet connection speed during the yoga session; the participants themselves having recorded the days of the week they practiced alone, as it does not guarantee they did it; the small sample size, which points to the need of replicating the study to corroborate these results and it is not possible to know whether the additional benefit of yoga was due to a placebo effect, although the literature in the topic shows that yoga is effective in reducing stress and improving well-being. However, the main limitation of the study is not having a control group that did not receive any intervention.

## Conclusion

Our study showed that both psychoeducation alone and combined with yoga can reduce the burden on family caregivers of people with Alzheimer's disease. The integration of online yoga practice with psychoeducation potentiated the improvement only in some aspects of quality of life and in the subjective perception of burden reduction. However, this additional benefit in burden was not objectively confirmed by the BI-Zarit scale. In conclusion, the integration of yoga with psychoeducation did not demonstrate significant additional benefits in reducing burden and improving the quality of life of family caregivers of people with Alzheimer's disease. Further randomized controlled clinical trials with a larger sample size and longer-term yoga programs are needed to better evaluate these results.

### Supplementary Information

Below is the link to the electronic supplementary material.

Supplementary file1 (DOCX 24 kb)

## Data Availability

The data that support the findings of this study are available from the corresponding author upon request.
