# Supplementary material for: Psychoeducation versus psychoeducation integrated with yoga for family caregivers of people with Alzheimer's disease: a randomized clinical trial
Source: Eur J Ageing. 2023 Nov 25;20(1):46. doi: 10.1007/s10433-023-00792-9 (PMC10676341; doi:10.1007/s10433-023-00792-9)
Supplement: Supplementary file 1 — Supplementary file1 (DOCX 24 kb) [file 10433_2023_792_MOESM1_ESM.docx]

**Supplementary Information**

**Psychoeducation vs. Psychoeducation integrated with Yoga for family caregivers of people with Alzheimer's disease: a randomized clinical trial**

**Journal: European Journal of Ageing**

### Edivaldo Lima de Araujo¹*, Marcos Rojo Rodrigues², Elisa Harumi Kozasa³, Shirley Silva Lacerda¹^,^³*

**¹ Faculdade Israelita de Ciências da Saúde Albert Einstein, Hospital Israelita Albert Einstein, São Paulo, Brazil**

**² Instituto de Ensino e Pesquisa em Yoga, São Paulo, Brazil**

**³ Hospital Israelita Albert Einstein, São Paulo, Brazil**

***Correspondence:**

Edivaldo Lima de Araujo

[edivaldo.dr@gmail.com](mailto:edivaldo.dr@gmail.com)

Shirley Silva Lacerda

[shirley.lacerda@einstein.br](mailto:shirley.lacerda@einstein.br)

.

**YOGA PROGRAM: CLASSES**

  The yoga exercises chosen for this work originate from the system known as *Hatha Yoga*, and among the techniques known in this system we highlighted the *Asanas* for our work.

The exercises were conducted as follows:

**Exercises from classes 1 and 2**

**1^st^ exercise:** stretching of the trunk region, mountain position (*parvatasana*).

Sitting comfortably with your spine erect, raise your arms laterally until your hands are above your head. Keep your elbows wide open (separated) and extend them, moving your joined hands towards the ceiling. Make the highest elevation possible, initially holding for 5 seconds and go back until you place your hands on your knees or thighs and your shoulders are very relaxed. At the beginning repeat the sequence three times and, as time goes by, increase the hold to 15 seconds doing it only once.

**2^nd^ exercise:** lateral inclination, wheel position (*chakrasana*) – adapted to the chair.

Sitting with your spine erect without touching the chair, raise your right arm laterally until it touches your right ear, extend your arm as if you were going to touch the ceiling with your hand. Lean to the left side until you feel a stretch in the region of your ribs on the right side. Hold for 10 seconds, come back and repeat on the other side. As the classes go on, the length of hold increases, if that is comfortable.

**3^rd^ exercise:** Stretching the neck muscles, the symbol of Brahma (*Brahma Mudra*)

Sitting comfortably with your spine erect, extend the cervical region in a movement identical to that of someone who is going to look up. Hold for three seconds, then return and flex your neck as someone who is going to look down. Hold for three more seconds, return to the center and then turn your head as if you wanted to look at the left side until your chin approaches the line of your left shoulder. Hold for three seconds longer, return to the center, turn your head to the other side and return to the center after three seconds. Initially we will do three repetitions of the complete sequence and later we will increase the time in each phase of the exercise, doing only one repetition.

**4^th^ exercise:** stretching of the posterior muscles of the lower limbs, the half pincer position (*ardha-paschimottanasana*) – adapted to the chair.

Sitting on the chair halfway forward with your right leg extended and your left leg bent with the sole of your foot on the floor, lean your torso forward trying to hold the tip of your right foot with your right hand. If you have difficulty holding your foot, you can hold your ankle. Hold initially for 10 seconds and increase the time with practice. Come back, rest for a few seconds and repeat on the other side.

**5^th^ exercise:** stretching of the trunk muscles, sitting twist (*Vakrasana*) – adapted to the chair.

Sitting with your spine straight without touching the back of the chair, place your right hand on your left hand and your left hand on the seat or back of the chair, whichever is more comfortable. With the help of your right hand pulling your left knee, twist your spine looking back over your left shoulder. Hold initially for 10 seconds and increase the time in subsequent classes if comfortable. Come back, rest observing the effect of the exercise and repeat it on the other side.

**6^th^ exercise**: seated spine extension, snake position (*Bhujangasana*) – adapted to the chair.

Sitting on the chair with your spine straight halfway forward, interlace your hands behind your back, extend your arms by bringing your shoulder blades together, expanding your chest, and looking up, stretching the front of your neck. Hold for 10 seconds, if it is comfortable, and gradually increase the time spent in the following classes.

**7^th^ exercise**: relaxation (*shavasana*) – adapted to the chair.

Sitting comfortably on the chair, feeling the support of your back on the backrest, your buttocks on the seat and your feet on the floor, observe the relaxation of all possible parts of your body. In the first classes, the teacher will lead the relaxation, suggesting the parts of the body to be observed and relaxed. With practice, the student will be in charge of his/her own relaxation.

**8^th^ exercise**: meditation (*pranadharana*) – sitting on the chair.

Sitting comfortably with your spine erect, observe your breathing without interfering with it. Breathing can be observed by the movements that occur in your abdomen or by the simple touch of air in your nostrils.

**Exercises from classes 3, 4 and 5**

**1^st^ exercise**: standing side bend (*chakrasana*).

Same as exercise 2 of the previous series, but standing up.

**2^nd^ exercise**: standing stretch, palm position (*talasana*).

Standing, feet parallel, almost together, pointing forward, raise both arms until they are next to your ears, extend your whole body, from the fingers to the tip of the toes. Stay initially for 10 seconds and increase the time in subsequent classes if comfortable.

**3^rd^ exercise**: standing balance, tree position (*vriksasana*)

Standing, feet parallel, almost together, raise your right leg, taking your right foot off the floor until you grab your right knee if possible and pull it towards your chest. Hold for 10 seconds if possible and increase in the following classes, if it is comfortable. Go back and repeat on the other side.

**4^th^ exercise**: posterior stretch, position of hands and feet together (*padahastasana*).

Standing with your feet approximately 20 cm apart, lean your torso forward as if you were going to touch the floor with your hands . Establish a comfortable limit and hold for 20 seconds trying to relax more and more. Knees can be semi-flexed if it is more comfortable.

**5^th^, 6^th^, 7^th^ and 8^th^ exercises** - the same as those corresponding to classes 1 and 2.

**Exercises from classes 6, 7 and 8**

**1^st^ exercise:** stretching of the upper trunk region (*lying talasana*).

Lying down with your legs bent and your arms along your body, raise your arms until your hands touch the ground behind your head, then extend your arms pleasantly as if stretching, come back in a slow and controlled movement and relax.

**2^nd^ exercise**:  posterior stretch, half plow variation (*ardha halasana*).

Lying down with your legs extended, bend your right leg, hold your right knee with both hands, pull it towards your chest and raise your left leg up to ninety degrees if possible. Hold for 10 seconds, come back and repeat on the other side.

**3^rd^ exercise:** posterior stretching with compression of the abdominal region (*pavanamuktasana*).

Lying down with your legs extended, bend your right knee, hold it with both hands, pull it towards your chest and bring your forehead closer to your bent knee,(ardha halasana).Hold for 10 seconds, go back, do it on the other side and then with both legs at the same time.

**4^th^ exercise:** lying twist (*kativrakasana*)

Lying down with your legs extended and your arms wide open, perpendicular to your torso, bend your right leg and rest the sole of your foot on top of your left knee. With your left hand, pull your right knee until it touches the floor or until you feel a stretch in the muscles of your lower back, gluteus muscles or sides of your left thigh. Hold for 10 seconds, come back, rest and do it on the other side.

**5^th^ exercise**: relaxation (*shavasana*) lying down.

Lying down comfortably with your legs extended or flexed (whichever is more comfortable), you can rest your head on a pillow if you prefer, observe your body part by part and then identify the feeling of your whole body relaxed. In the beginning, the teacher leads and then the student can lead his/her own relaxation.
